# Pericardial synovial sarcoma: radiological findings

**DOI:** 10.1590/0100-3984.2016.0200

**Published:** 2018

**Authors:** Diogo Costa Leandro de Oliveira, Eduardo Oliveira Pacheco, Larissa Teixeira Ramos Lopes, Claudio Calazan do Carmo, Alessandro Severo Alves de Melo

**Affiliations:** 1 Universidade Federal Fluminense (UFF), Niterói, RJ, Brazil.; 2 Hospital Niterói D’Or, Niterói, RJ, Brazil.; 3 Grupo Oncologia D’Or, Niterói, RJ, Brazil.

*Dear Editor,*

**Figure 1:**
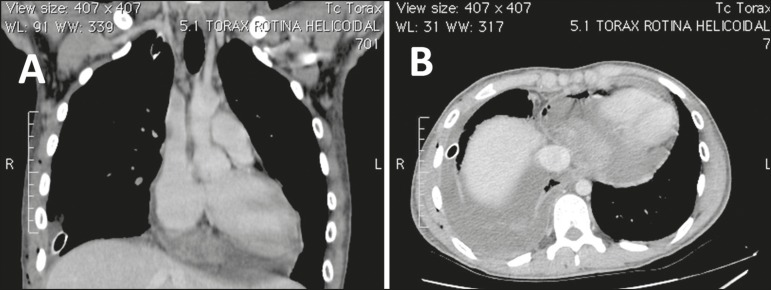
Contrast-enhanced chest CT. Coronal (**A**) and axial (**B**) slices in a mediastinal window, showing a solid heterogeneous mass with contrast enhancement in the posterior portion of the pericardial sac associated with pericardial effusion and pleural effusion on the right, together with pleural drainage.

**Figure 2:**
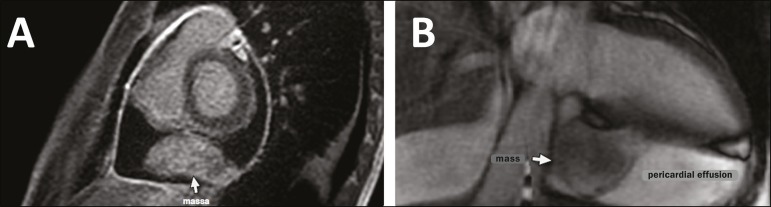
Cardiac MRI with delayed enhancement, in short-axis and long-axis views (**A** and **B**, respectively), showing a solid mass with a heterogeneous content, measuring 3.2 × 6.1 × 3.9 cm, in the posterior portion of the pericardial sac with adhesion points, with significant heterogeneous contrast uptake (as determined by the delayed enhancement technique), as well as pericardial inflammation.

An 18-year-old male patient was admitted to the hospital with a 15-day history of cough and fatigue. Cardiac auscultation revealed muffled heart sounds. A chest X-ray obtained at admission showed an increase in the cardiac silhouette and moderate pleural effusion on the right. An echocardiogram was performed, which demonstrated a significant pericardial effusion with signs of diastolic restraint and a rounded, hypoechoic mass with regular contours, measuring 3.6 × 3.9 cm, located posterior to the right atrium. The patient underwent pericardiocentesis, with analysis of the fluid collected. Computed tomography (CT) of the chest and cardiac magnetic resonance imaging (MRI) were requested. The chest CT (Figures 1) revealed a solid, heterogeneous mass with contrast enhancement in the posterior portion of the pericardial sac, associated with pericardial effusion and pleural effusion. In the cardiac MRI (Figure 2), a solid mass with a heterogeneous content measuring 3.2 × 6.1 × 3.9 cm was observed in the posterior portion of the pericardial sac, with adhesion points and significant heterogeneous contrast uptake (as determined by the delayed enhancement technique), as well as pericardial inflammation. Based on the imaging findings suggestive of neoplasia and the inconclusive pericardial fluid cytologic findings, we decided to perform surgical resection of the mass. The histopathological examination of the surgical specimen resulted in a diagnosis of synovial sarcoma. After one month of hospitalization, the patient was discharged to oncology outpatient follow-up.

Cardiac MRI has taken on an ever-increasing role in the study of cardiovascular diseases^(^^1^^-^^4^^)^. Pericardial synovial sarcoma is a primary malignant tumor of the pericardium that is histologically similar to the synovium and originates from mesenchymal cells^(^^5^^)^. It is an extremely rare disease, the exact prevalence of which remains unknown, with a slight predilection for young males^(^^6^^)^. The symptoms range from none to pericardial effusion with cardiac tamponade, dyspnea, fever, weight loss, and embolic phenomena^(^^7^^)^. Although the prognosis for pericardial synovial sarcoma is poor, some patients may benefit from surgical resection and radiotherapy, with or without chemotherapy^(^^8^^,^^9^^)^. In asymptomatic patients, the working diagnosis is based on incidental findings of lesions in cardiac imaging, whereas it is based on the findings of directed imaging tests in symptomatic patients; in either case, the diagnosis can be confirmed only through histopathological analysis^(^^6^^,^^8^^)^.

Although the tumor image is nonspecific on the echocardiogram of an individual with pericardial synovial sarcoma, it is fundamental for the initial detection of the disease, quantification of the pericardial effusion, evaluation of cardiac function, and evaluation of cardiac restraint, as well as allowing comparative analysis with sequential follow-up examinations^(^^10^^)^. A solid, heterogeneous mass, with multilocular areas^(^^11^^)^ and internal septa, is observed on CT and MRI; in some cases, there is invasion of adjacent structures, pericardial effusion and foci of metastases. Cardiac MRI is considered the best modality for the detection and characterization of pericardial synovial sarcoma, because it makes it possible to observe the degree of vascularization, to better detail the cardiac invasion, and to monitor the post-treatment evolution^(^^10^^,^^12^^)^. In this context, it can be concluded that, although the imaging tests do not confirm the diagnosis, they play a fundamental role in the detection and characterization, as well as in the preoperative and postoperative planning, of pericardial synovial sarcoma.
